# Alanine racemase is essential for the growth and interspecies competitiveness of *Streptococcus mutans*

**DOI:** 10.1038/ijos.2016.34

**Published:** 2016-10-14

**Authors:** Yuan Wei, Wei Qiu, Xue-Dong Zhou, Xin Zheng, Ke-Ke Zhang, Shi-Da Wang, Yu-Qing Li, Lei Cheng, Ji-Yao Li, Xin Xu, Ming-Yun Li

**Affiliations:** 1 State Key Laboratory of Oral Diseases, West China Hospital of Stomatology, Sichuan University, Chengdu, China; 2 Department of Operative Dentistry and Endodontics, West China Hospital of Stomatology, Sichuan University, Chengdu, China

**Keywords:** alanine racemase, biofilm, D-alanine, dental caries, *Streptococcus mutans*

## Abstract

D-alanine (D-Ala) is an essential amino acid that has a key role in bacterial cell wall synthesis. Alanine racemase (Alr) is a unique enzyme that interconverts L-alanine and D-alanine in most bacteria, making this enzyme a potential target for antimicrobial drug development. *Streptococcus mutans* is a major causative factor of dental caries. The factors involved in the survival, virulence and interspecies interactions of *S. mutans* could be exploited as potential targets for caries control. The current study aimed to investigate the physiological role of Alr in *S. mutans*. We constructed *alr* mutant strain of *S. mutans* and evaluated its phenotypic traits and interspecies competitiveness compared with the wild-type strain. We found that *alr* deletion was lethal to *S. mutans*. A minimal supplement of D-Ala (150 μg·mL^−1^) was required for the optimal growth of the *alr* mutant. The depletion of D-alanine in the growth medium resulted in cell wall perforation and cell lysis in the *alr* mutant strain. We also determined the compromised competitiveness of the *alr* mutant strain relative to the wild-type *S. mutans* against other oral streptococci (*S. sanguinis* or *S. gordonii*), demonstrated using either conditioned medium assays or dual-species fluorescent *in situ* hybridization analysis. Given the importance and necessity of *alr* to the growth and competitiveness of *S. mutans*, Alr may represent a promising target to modulate the cariogenicity of oral biofilms and to benefit the management of dental caries.

## Introduction

D-alanine (D-Ala) is an essential substance for the synthesis of peptidoglycan (PG), an important constituent of the cell wall of both Gram-negative and Gram-positive bacteria.^1, 2^ The synthesis of the cell wall is initiated with uridine diphosphate (UPD)-N-acetylmuramic acid, L-Ala, D-glutamic acid (D-Glu) and meso-diaminopimelic acid to generate UDP-N-acetylmuramyl-L-Ala-D-Glu-meso-diaminopimelate.^1, 3^ A D-Ala dipeptide is coupled to this intermediate through UDP-N-acetylmuramyl-tripeptide-D-Ala-D-Ala ligase to generate UDP-N-acetylmuramyl-tripeptide-D-Ala-D-Ala, which is subsequently incorporated into the growing PG peptide chain. The D-Ala dipeptide is synthesized by two enzymes: alanine racemase (Alr), which converts L-enantiomers of alanine to the D-counterparts, and D-Ala-D-Ala ligase, which generates the D-Ala dipeptide.^3^

Alr is a unique enzyme responsible for the racemization of L-Ala to D-Ala.^4^ Previous studies have revealed that Alr is essential for the survival of numerous Gram-positive bacteria, including *Bacillus, Lactobacillus*, *Burkholderia pseudomallei, Burkholderia mallei* and *Mycobacterium tuberculosis*. Knockout of the *alr* gene in these bacteria resulted in a strict exogenous D-Ala-dependent growth phenotype.^5, 6, 7, 8^ Similar growth arrest and extensive cell lysis were also observed in the *alr* mutant of Gram-negative *Escherichia coli*, which underwent rapid and extensive cell lysis when deprived of D-Ala.^5, 9^ Further *in vitro* and *in vivo* studies of *M. tuberculosis* have shown that Alr is a primary target of D-cycloserine, and the inhibition of Alr alone could reduce the viability and perseverance of this bacterium.^8^

*Streptococcus mutans* is the major caries-associated bacterium in humans. During cariogenic conditions (e.g., frequent sugar intake), *S. mutans* metabolizes carbohydrates, leading to acid accumulation and subsequent fall in pH in the dental biofilm.^10^ The acidic micro-environment selectively enriches acidogenic/aciduric species (e.g., mutans streptococci and lactobacilli) and suppresses less aciduric commensal residents (e.g., *Streptococcus sanguinis* and *Streptococcus gordonii*). This microbial disequilibrium leads to continuous pH decline to reach a critical pH, below which tooth hard-tissue demineralization begins and dental caries gradually occur.^11, 12^ Although Alr has been well documented as closely associated with the viability and survival of various bacteria,^5, 6, 7, 8, 9^ its physiological importance in *S. mutans* has not been explored, particularly in a biofilm context. In the present study, we constructed *alr* mutant strain and investigated the physiological role of *alr* in the cell growth, cell wall integrity and interspecies competitiveness of *S. mutans.* We found that *alr* is an essential factor to maintain the growth and cell wall integrity of *S. mutans*. Deletion of *alr* in *S. mutans* significantly compromised its competitiveness with other co-residents (e.g., *S.* *sanguinis*) in the oral biofilm. We concluded that Alr might represent a promising drug target to modulate the cariogenicity of oral biofilm and to benefit the management of dental caries.

## Materials and methods

### Bacterial strains, growth media and reagents

*S. mutans* UA159 was obtained from the Dental Research Institute, University of Toronto^13^ and was routinely anaerobically (90% N_2_, 5% CO_2_, 5% H_2_) or aerobically (95% air, 5% CO_2_) incubated at 37 °C in brain heart infusion (BHI) broth (Difco, Sparks, MD, USA). For the transformation experiments, the cells were maintained in Todd-Hewitt medium (Difco, Sparks, MD, USA) supplemented with 3 ġL^−1^ yeast extract (THYE; Difco, Sparks, MD, USA). The competence-stimulating peptide used for *S. mutans* transformation was custom-synthesized by Sangon Biotech (Shanghai, China). For the selection of antibiotic-resistant colonies, BHI plates were supplemented with erythromycin (erm, 12.5 μġmL^−1^). D-Ala (150 μg·mL^−1^) was added to the BHI broth to promote the growth of the *S. mutans alr* mutant strain. The optical density (OD) of the cell culture was measured at 600 nm (OD_600_).

Taq DNA polymerase, restriction enzymes and T_4_ DNA ligase were all purchased from New England Biolabs (Ipswich, MA, USA). Taq DNA polymerase was used for overlapping polymerase chain reaction (PCR).

### Construction of the *alr* mutant strain

**Figure 1:**
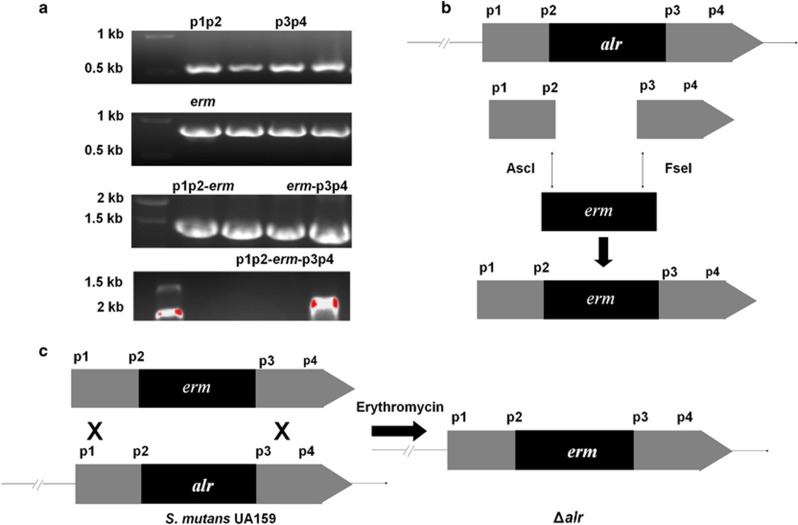
**The *S. mutans alr* mutant was constructed using homologous recombination**. (**a**) Two 500 bp fragments were generated (p1p2: up-stream, and p3p4: down-stream of *alr*) using the primer pair *alr*-up-f/*ldh* and *alr*-down-f/*ldh*. An *erm* fragment (876 bp) was amplified using the primer pair *ldh*-f/*erm*-r. (**b**) The three digested fragments ligated using T_4_ DNA ligase to generate the proposed segment. (**c**) This fragment was transformed into *S. mutans*, and the transformants were selected on BHI broth plates containing 12.5 μg·mL^−1^ of erythromycin. Δ*alr, S. mutans alr* mutant; BHI, brain heart infusion.

The primers used in this study are shown in Table 1. Two 500 bp fragments (up- and down-stream of *alr*) were generated through PCR using the primer pair *alr*-up-f/*ldh* and *alr*-down-f/*ldh*. The *erm* fragment (876 bp) was amplified with primer pair *ldh*-f/*erm*-r by PCR.^14^ The three pairs of primers were specifically designed for subsequent ligation based on the sequences adjacent to mutagenesis site. The restriction enzymes *Asc*I (New England Biolabs, Ipswich, MA, USA) and *Fse*I were used to digest the up- and down-stream fragments, respectively, and the aforementioned enzymes were also used to digest the purified *erm* segment. The three digested fragments were subsequently mixed, and T4 DNA ligase was added to generate the proposed segment (Figure 1).^15, 16, 17^ The resulting 1.876 kb fragment was transformed into *S. mutans*, and transformants were selected on BHI plates containing 12.5 μg·mL^−1^ of erythromycin. The *alr* deletion mutant was confirmed using sequencing. All primers used are listed in Table 1.

### Growth of the *alr* mutant

*S. mutans* UA159 and the *alr* mutant strain were cultivated overnight in BHI broth. Stationary phase cultures were diluted 1:20 in BHI broth and incubated at 37 °C until the OD_600_ reached 0.2. A 20 μL aliquot of the cell culture and 180 μL of BHI broth were added to each well of a 96-well plate. The OD of the bacteria culture was measured at intervals over a period of 1 h. The cells were diluted to 1 × 10^6^ CFU·mL^−1^, plated onto BHI broth agar plates, and incubated at 37 °C for 24 h.

### Transmission electron microscopy

Transmission electron microscopy (TEM) was performed as previously described.^18^ Approximately 10 mL of cell culture was harvested by low-speed centrifugation (3 000*g*, 10 min), washed twice in 200 mmol̇L^−1^ sodium cacodylate buffer, pre-fixed with 2.5 ġL^−1^ glutaraldehyde and fixed with 10 ġL^−1^ OsO_4_. Samples were embedded in Epon resin, and thin sections (60 nm) were prepared using a microtome. The sections were stained with 40 ġL^−1^ uranyl acetate and subsequently with 4 ġL^−1^ lead citrate and were examined using a Tecnai G2 F20 S-TWIN electron microscope (FEI, Hillsboro, OR, USA).

### Conditioned media assay

To investigate whether other commensal colonizers such as *S. sanguinis* and *S. gordonii* can support the growth of the *alr*-deficient mutant under exogenous D-Ala-deprived conditions, we performed a conditioned media assay as previously described.^19^ Briefly, the culture supernatants of *S. sanguinis* and *S. gordonii* at the mid-exponential phase were collected and filter sterilized as a conditioned medium for the growth of the *alr* mutant. After aerobic incubation (5% CO_2_) for 24 h, the OD_600_ values of the bacterial cultures were determined to evaluate the effect of conditioned medium on the growth of the *alr* mutant. We also diluted the conditioned medium 1:2 with fresh BHI broth medium to evaluate the dose-dependent effects of conditioned medium. In addition, an exogenous D-amino acid oxidase (0.5 U·mL^−1^, Sigma, St Louis, MO, USA) was added to the conditioned medium to investigate whether the growth compensatory effects of conditioned medium on the *alr* mutant could be attenuated.

### Fluorescent *in situ* hybridization

Fluorescent *in situ* hybridization (FISH) was performed according to Zheng *et al.*^19^ Biofilm specimens established on saliva-coated glass coverslips were rinsed with distilled water and dried for 10 min at 46 °C. To enhance probe penetration, the specimens were treated with 1 mL of lysis buffer (100 mmol̇L^−1^ tris(hydroxymethyl)aminomethane (Tris)-HCl, 50 mmol̇L^−1^ ethylene diamine tetraacetic acid, and 30 mg·mL^−1^ lysozyme (Sigma), pH 8.0) for 20 min at 37 °C. The specimens were subsequently rinsed with distilled water, serially dehydrated in ethanol (50%, 80% and 96% 3 min each), dried for 10 min at 46 °C, exposed to 20 μL of hybridization buffer (0.9 mol̇L^−1^ NaCl, 20 mmol̇L^−1^ Tris-HCl, 0.01% sodium dodecyl sulfate (SDS) and 20% formamide) containing the designated oligonucleotide probes (2 nmol̇L^−1^), and incubated at 46 °C for 90 min. After hybridization, the specimens were washed in buffer (20 mmol̇L^−1^ Tris-HCl, 0.01% SDS, 5 mmol̇L^−1^ EDTA and 215 mmol̇L^−1^ NaCl) for 15 min at 48 °C in a water bath and were subsequently rinsed in ice-cold nuclease-free water.

The specific probe for *S. sanguinis* was designed using ARB software (Linux release arb_5.3, Bremen, Germany). The *S. mutans-*specific probe was synthesized according to the sequence provided in a previous study.^20^ The probe sequences are listed in Table 2. Biofilms were examined using an Olympus BX3-CBH fluorescence microscope (Olympus, Tokyo, Japan) with an SpGr-B Filter (Semrock, Rochester, NY, USA) for Alexa Fluor 488 and an SpRed-B Filter (Semrock, Rochester, NY, USA) for Alexa Fluor 594. Black-and-white micrographs from at least five randomly selected positions of each sample were obtained using an Andor iXon3 camera (Andor Technology, Beijing, China) and were processed using Cell Sens Dimension (Olympus, Tokyo, Japan) and Photoshop CS 4.0 (Adobe, San Jose, CA, USA) without any qualitative changes to the raw images. The ratio of *S. mutans/S. sanguinis* was calculated based on the coverage area of each bacterium analysed using Image Pro Plus 6.0 (Media Cybernetics, Silver Spring, MD, USA).

### Statistical analysis

All experiments were performed in triplicate. The statistical analysis was performed using SPSS16.0 software (SPSS, Chicago, IL, USA). Differences were considered as significant when the two-tailed *P*-value was <0.05.

## Results

### Growth of the *alr*- mutant is strictly dependent on exogenous D-Ala

**Figure 2:**
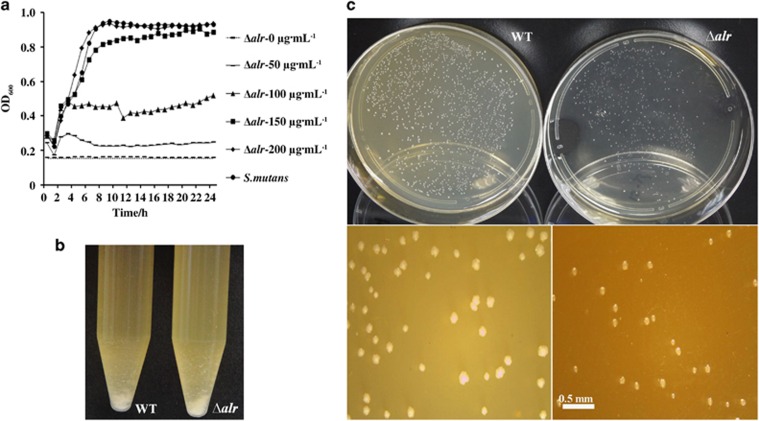
**Phenotypic traits of the *alr* mutant compared with *S. mutans***. (**a**) Growth curve of *S. mutans* and the *alr* mutant in the presence of an increasing dose of D-Ala for 24 h (0–200 μg·mL^−1^). (**b**) Growth of planktonic cultures of *S. mutans* (left) and the *alr* mutant strain (right) at the exponential phase in tubes. No significant difference was observed. (**c**) Representative stereoscopic micrographs of colonies of *S. mutans* and the *alr* mutant strain grown on agar plates. The results were averaged from three independent experiments, shown as the mean±standard deviation. Ala, alanine; Δ*alr*, *S. mutans alr* mutant; WT, wild-type *S. mutans*.

As shown in Figure 2a, *alr* deletion without the addition of exogenous D-Ala was lethal to *S. mutans*. The optimal growth of the *alr* mutant strain was observed after adding no less than 150 μg·mL^−1^ of D-Ala to the BHI medium (Figure 2a).

In addition, no significant difference with respect to the planktonic culture (Figure 2b), colony shape, colour and transparency (Figure 2c) of the *alr* mutant strain was observed relative to that of the wild-type UA159 strain, although the colony size of the *alr* mutant strain was relatively smaller (Figure 2c).

### D-Ala starvation causes cell morphology alterations of the *alr* mutant

**Figure 3:**
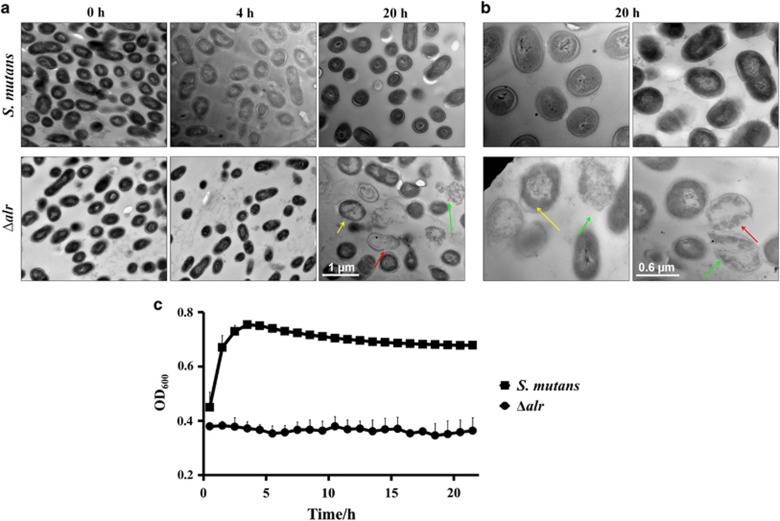
**Morphological alterations and growth of the *alr* mutant under D-Ala starvation**. (**a**, **b**) Representative transmission electron micrographs of *S. mutans* and *alr* mutant strain (Δ*alr*) under D-Ala starvation for 0, 4 and 20 h. The red arrows indicate plasmolysis, and the green arrows indicate damaged cells. (**c**) The growth curve of *S. mutans* and Δ*alr* under D-Ala starvation. The results were averaged from three independent experiments, presented as the mean±standard deviation. Ala, alanine; Δ*alr*, *S. mutans alr* mutant.

To determine how *alr* deletion affects the growth of *S. mutans*, we further examined the kinetic cell morphological alterations of the *alr* mutant during D-Ala depletion using TEM. As shown in Figure 3a, when incubated in BHI without exogenous D-Ala, initial D-Ala starvation (4 h) did not result in significant morphological changes in the *alr* mutant relative to those of the wild-type control. However, the *alr* mutant strain exhibited pronounced morphological alterations after prolonged D-Ala starvation (20 h). Specifically, conspicuous cell lysis was observed in the *alr* mutant strain relative to the wild-type control (Figure 3b). These morphological alterations indicate the pronounced cell lysis of the *alr* mutant, potentially reflecting compromised cell wall stability and integrity after the deletion of *alr* in a D-Ala-deprived environment.

Consistent with increased cell lysis of the *alr* mutant, kinetic monitoring of the bacterial growth in the BHI medium without exogenous D-Ala also showed the significant growth arrest of the *alr* mutant, reflected by the unchanged OD_600_ values compared with the wild-type strain (Figure 3c).

### Deletion of *alr* in *S. mutans* compromises its interspecies competitiveness

**Figure 4:**
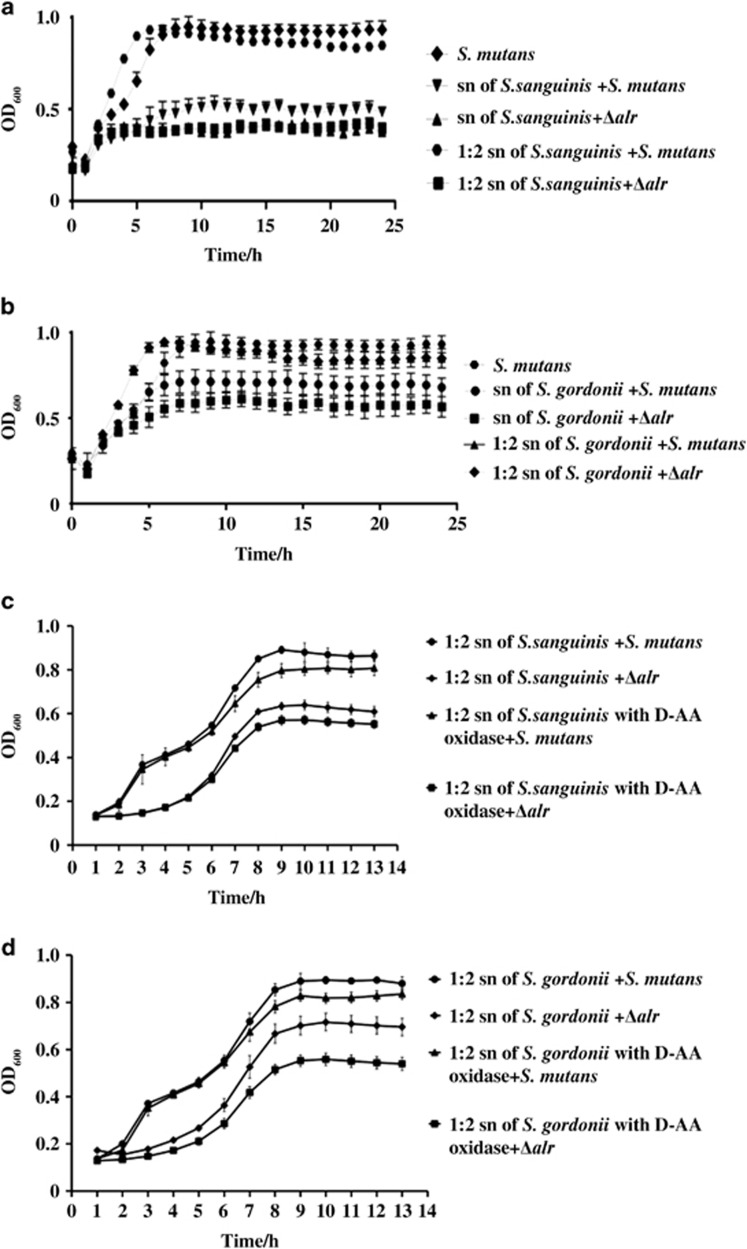
**Effect of conditioned medium derived from other commensal bacteria on the growth of the *alr* mutant**. (**a,** **b**) Effect of *S. sanguinis*-derived and *S. gordonii*-derived conditioned medium on the growth of the Δ*alr* with or without exogenous D-Ala, respectively. (**c**, **d**) Effect of *S. sanguinis*-derived and *S. gordonii*-derived conditioned medium supplemented with D-AA oxidase on the growth of Δ*alr*. The results were averaged from three independent experiments and presented as the mean±standard deviation. “sn”: supernatant from *S. sanguinis* or *S. gordonii* culture; “1:2 sn”: 1:2 diluted supernatant with fresh BHI broth media. AA, amino acid; Ala, alanine; Δ*alr*, *S. mutans alr* mutant; BHI, brain heart infusion.

We further investigated the interspecies competitiveness of the *alr* mutant against other common oral streptococci, such as *S. sanguinis* and *S. gordonii*. We observed that the *alr* mutant strain could survive in the conditioned media of *S. sanguinis* or *S. gordonii* without exogenous D-Ala (Figure 4a and 4b), although its growth in the conditioned media was significantly inhibited relative to that of the wild-type parental strain. The addition of D-amino acid oxidase into the conditioned media further attenuated the growth of *alr* mutant, but had no significant effect on the growth of the wild-type *S. mutans* strain (Figure 4c and 4d). These data suggest that although the *alr* mutant might feed on the metabolites derived from other oral streptococci (D-Ala) to partially compensate for the scarce endogenous D-Ala, its perseverance in the co-existence of other oral streptococci remained compromised.

**Figure 5:**
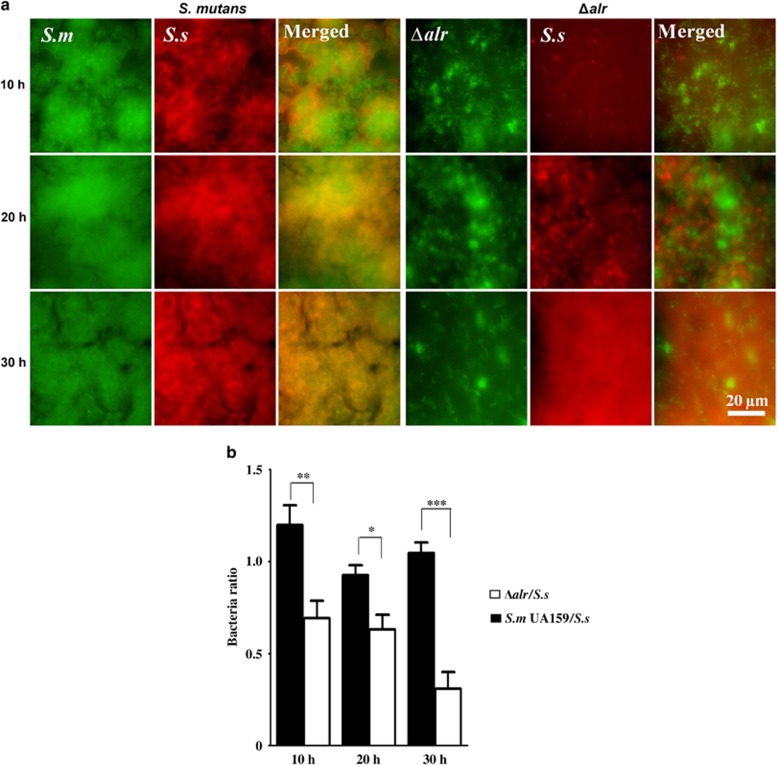
**Interspecies competition between *alr* mutant and *S. sanguinis* in dual-species biofilms**. (**a**) *S. mutans* (green), *alr* mutant strain (Δ*alr*, green) and *S. sanguinis* (red) were labelled using species-specific fluorescent *in situ* hybridization probes. (**b**) The ratio of *S. mutans/S. sanguinis* quantified using the coverage area of each species. The results were averaged from at least five randomly selected positions of each sample and shown as the mean±standard deviation. *P<0.05, **P<0.01, ***P<0.0001. Δ*alr, S. mutans alr mutant; S.m, S. mutans; S.s, S. sanguinis*.

To further demonstrate the role of *alr* in the interspecies competition between *S. mutans* and *S. sanguinis* within biofilm, we used species-specific FISH to quantify the bacterial composition in a dual-species biofilm model (Figure 5a). Consistent with the data obtained from conditioned media assays, the colonization of the *alr* mutant was significantly reduced, as reflected by a significantly lower *S. mutans*/*S. sanguinis* ratio within the dual-species biofilm after 30 h compared with that of the wild-type *S. mutans* controls at corresponding time points (Figure 5b). These data further support that *alr* is critical for the interspecies competitiveness of *S. mutans* within biofilm.

## Discussion

Alr is responsible for the production of D-Ala, which is used for PG biosynthesis in all bacteria, including harmful pathogens.^1, 2, 21^ Alr is encoded by *alr*, which is ubiquitous in prokaryotes but absent in eukaryotes, making this enzyme a great target for antimicrobial drug development. Previous knockout studies have shown that bacteria deficient in the *alr* gene require an external source of D-Ala for survival,^6, 7, 8, 9, 22^ further supporting the idea that *alr* could be a feasible target for antimicrobial drugs. In the present study, deleting the *alr* gene in *S. mutans*, a major caries-associated bacterium in the oral cavity of humans, revealed that the viability and growth of the *alr* mutant was strictly dependent on exogenous D-Ala derived from either the growth media or other commensal co-colonizers.

The observed kinetic growth of the *alr* mutant under D-Ala starvation up to 24 h is similar to that of the previously reported *alr* mutant of *Lactobacillus plantarum.*^23^ The relative static cell amount during D-Ala starvation indicates balanced cell lysis and proliferation. We speculated that dead cells under D-Ala starvation might not completely rupture, reflecting either osmo-protection in the nutrient-rich growth medium or the protection of relatively thick and rigid cell wall of *S. mutans*. In addition, the *alr*-deficient cells might feed on the recycled D-Ala from the cell wall of the lysed cells, thus maintaining a balanced cell mass under D-Ala starvation.

The pronounced cell lysis of the *alr* mutant was observed under D-Ala-deprived conditions. Cell lysis was also observed in the *alr* mutant of *L. plantarum* and *E. coli,*^6, 9^ reflecting the disturbed balance between PG degradation and biosynthesis during septation after the inactivation of Alr.^23, 24^ The PG peptide stem often contains D-Ala residues at the fourth and fifth positions.^25^ The disruption of the synthesis of these two residues could lead to the cell death of Gram-positive *Bacillus* and *Lactobacillus.*^6, 26^ In Gram-positive bacteria, such as *Bacillus subtilis* and *Staphylococcus* *aureus*, the inhibition of the D-alanylation on teichoic acid (TA) reflects the inactivation of *dlt* operon-stimulated autolysis.^27, 28^ The removal of D-alanyl residues from TA could result in the formation of cell surface holes, which likely confer a higher affinity to cationic PG hydrolases and thereby facilitating cell lysis.^27^ The observed cell wall damage and cell lysis of the *alr* mutant in the current study might also reflect the defective PG peptide stem and the D-alanylation of TA. The glutamate racemase (*murl*) has also been reported as an essential enzyme in the synthesis of the bacterial cell wall.^29, 30^ The *murl* mutant of *E. coli* lysed in the absence of D-glutamic acid,^31^ and the *murl* mutant of *Mycobaterium* *smegmatis* also showed aberrant cell shapes.^32^ Although the precise mechanisms remain unknown, the deletion of *alr* not only led to growth arrest but also to cell death, indicating that *alr* might be a potential bactericidal target for the inhibition of *S. mutans*.

Interestingly, we also observed that the *alr* mutant of *S. mutans* could survive in concert with other common oral streptococci, such as *S. sanguinis* and *S. gordonii*. The partially recovered growth of the *alr-*deficient mutant in conditioned medium from either *S. sanguinis* or *S. gordonii* could be further attenuated through D-amino acid oxidase. These results and data showing that the *alr* mutant requires exogenous D-Ala for survival, indicating that although the *alr* mutant might feed on either exogenous D-Ala or bacterial metabolites from other co-residents, the growth and competitiveness of the *alr* mutant strain are significantly compromised compared with the parental strain. This finding further supports the hypothesis that *alr* might be a promising target to control the prevalence of cariogenic *S. mutans* in a multispecies microbial consortium. Because Alr is universal in all bacteria, the utilization of this enzyme to develop antimicrobial drugs specific for *S. mutans* in multispecies microbial consortium is worthy of further investigation. Strategies as proposed by Professor Shi's group^33, 34, 35^ might be used to link Alr small-inhibitors with a targeting peptide (e.g., Competence stimulating peptide) and to achieve *S. mutans*-specific killing, even in a complex microbial consortium.

Taken together, the results of the present study provide the first evidence that *alr* is critical for the viability, growth and interspecies competitiveness of *S. mutans*. Given the ecological importance of *S. mutans* in initiating oral microbial disequilibrium and dental caries, Alr could be exploited as an antimicrobial target for *S. mutans*, thereby contributing to the control of dental caries.

## Figures and Tables

**Table 1:** Oligonucleotide primers used for the construction of the *S. mutans alr* mutant

Primers	Nucleotide sequence (5′-3′)
*alr*-up-f	ATCCCATGAACATCAGTTTATGTC
*alr*-up-*ldh*	**GGCGCGCC**CTAGCAATCATAAGTTATTCTCCTCC
*alr*-down-r	**GGCCGGCC**GTTAAGTGGCTGAACTTTTTTGGC
*alr*-down-*erm*	CTGATCAAGCTCCACCTTATCTTG
*Idh*-f	**GGCGCGCC**CCGGGCCCAAAATTTGTTTGAT
*erm*-r	**GGCCGGCC**AGTCGGCAGCGACTCATAGAAT

The nucleotide sequence indicated in bold and underlined is the restriction enzyme site of AscI, and the sequence indicated in bold is the restriction enzyme site of FseI.

**Table 2:** Oligonucleotide probes used for fluorescent *in situ* hybridization

Probes	Nucleotide sequence (5′-3′)	Target gene
*S. mutans*	(Alexa Fluor 488)-ACTCCAGACTTTCCTGAC	16S rRNA
*S. sanguinis*	(Alex Fluor 594)-GCATACTATGGTTAAGCCACAGCC	16S rRNA
